# Taste and smell function in long-term survivors after childhood medulloblastoma/CNS-PNET

**DOI:** 10.1007/s00520-022-07048-9

**Published:** 2022-04-15

**Authors:** Kristine Eidal Tanem, Einar Stensvold, Petter Wilberg, Anne B. Skaare, Preet Bano Singh, Petter Brandal, Bente Brokstad Herlofson

**Affiliations:** 1 Dep. of Oral Surgery and Oral Medicine, Faculty of Dentistry, University of Oslo, Oslo, Norway; 2 Dep. of Pediatrics, Oslo University Hospital, Oslo, Norway; 3 The Faculty of Medicine, Institute of Clinical Medicine, University of Oslo, Oslo, Norway; 4 Oral Health Centre If Expertise in Eastern Norway (OHCE), Oslo, Norway; 5 Dep. of Pediatric Dentistry and Behavioral Science, Faculty of Dentistry, University of Oslo, Oslo, Norway; 6 Dep. of Oncology, Oslo University Hospital, Oslo, Norway; 7 Section for Cancer Cytogenetics, Institute for Cancer Genetics and Informatics, Oslo University Hospital, Oslo, Norway; 8 Unit of Oral and Maxillofacial Surgery, Division for Head, Neck, and Reconstructive Surgery, Dep. of Otorhinolaryngology, Oslo University Hospital, Oslo, Norway

**Keywords:** Brain tumor, Childhood cancer survivors, Late effects, Taste function, Smell function

## Abstract

**Purpose:**

To investigate taste and smell function in survivors, with a minimum of 2 years since treatment of childhood medulloblastoma (MB)/central nervous system supratentorial primitive neuroectodermal tumor (CNS-PNET).

**Methods:**

This cross-sectional study included 40 survivors treated ≤ 20 years of age. Taste strips with four concentrations of sweet, sour, salt, and bitter were used to assess taste function in all participants. Score from 0 to 16; ≥ 9 normogeusia, < 9 hypogeusia, and complete ageusia which equals no sensation. No sensation of a specific taste quality equals ageusia of that quality. Thirty-two participants conducted smell testing using three subtests of Sniffin’ sticks: threshold, discrimination, and identification. Together they yield a TDI-score from 1 to 48; functional anosmia ≤ 16.00, hyposmia > 16.00– < 30.75, normosmia ≥ 30.75– < 41.50, and ≥ 41.50 hyperosmia. Results were compared with normative data. Survivors rated their taste and smell function using a numerical rating scale (NRS) score 0–10.

**Results:**

Forty survivors with a mean time since treatment of 20.5 years, 13 (32.5%) were diagnosed with hypogeusia, nine (22.5%) of these being ageusic to one or more taste qualities. Seventeen (53%) of 32 participants were diagnosed with hyposmia. The mean scores of the olfactory subtests, and TDI score were significantly lower than normative data (*P* < 0.0001). The mean NRS scores of smell and taste function were 7.9 ± 1.5 and 8 ± 1.3, respectively.

**Conclusion:**

Our study showed impaired taste and smell function in survivors of childhood MB/CNS-PNET using objective measurements. However, subjective ratings did not reflect objective findings.

**Supplementary information:**

The online version contains supplementary material available at 10.1007/s00520-022-07048-9.

## Introduction

The embryonal tumors medulloblastoma (MB) and central nervous system supratentorial primitive neuroectodermal tumor (CNS-PNET) are malignant childhood brain tumors [1, 2]. MB is located in the infratentorial brain and CNS-PNET in the supratentorial brain [1, 2]. Both entities are treated similarly with a multidisciplinary approach involving surgery, radiotherapy (RT), and/or chemotherapy [2–4]. Due to the high risk of severe neurocognitive impairment, patients under the age of 3–5 years are treated without RT in most countries [4, 5]. Although survival rates have improved [6], survivors of childhood MB/CNS-PNET may experience several complications and long-term effects such as posterior fossa syndrome, second primary neoplasm, hearing and visual impairment, cerebrovascular disease, and endocrinopathies [1, 5, 7, 8].

Reduced or altered taste and smell function are possible long-term effects of cancer treatment [9] and may have severe impact on patients’ diet, nutritional status, and health maintenance [9, 10], as well as quality of life [11]. Changes in taste and smell may be present before, during, or after cancer treatment [9]. Most studies have investigated alterations during treatment [9, 12, 13], while less research has focused on taste and smell function years after treatment [9]. Taste buds have a lifespan of approximately 10 days and are continuously replaced [14], while the olfactory neurons regenerate every 3–6 months [14]. Evidence regarding recovery of chemosensory function in cancer survivors is conflicting [9].

Irreversible taste changes after RT in head-and neck cancer (HNC) patients are well known [10]. Taste impairment has been reported even when the irradiation field does not directly involve the oral cavity [15]. Smell function may also be impaired in HNC patients [16, 17]. Few studies have addressed taste and smell function in survivors treated for other malignancies than HNC [18–21], and to our knowledge only a few have included CNS cancers like MB/CNS-PNET [18, 20, 21].

As most MB/CNS-PNET patients are treated with craniospinal irradiation (CSI) [2–4], there is a risk of damaging healthy tissue in the head and neck region where taste and smell receptors are located [14, 22]. Johannesen and coworkers (2002) reported taste impairment in three out of 33 brain tumor survivors treated with RT [20]. Leyrer and coworkers (2013) assessed taste and smell dysfunctions in patients after brain RT using a validated questionnaire [21]. They reported that 14 out of 20 patients experienced taste dysfunction and 10 out of 20 patients had smell impairment [21]. When adding chemotherapy to the treatment of MB/CNS- PNET patients, the risk of chemosensory damage may increase [9, 23].

Due to limited studies on taste and smell function in survivors of CNS cancers, especially in pediatric survivors, the aim of this study was to investigate objective and subjective taste and smell function in long-term survivors after childhood MB/CNS-PNET.

## Material and methods

### Patients/study design

This cross-sectional study on taste and smell function was part of a large regional multidisciplinary study investigating health impairments in survivors of pediatric MB/CNS-PNET [3, 24]. Participants had to (1) be treated at Oslo University Hospital (OUH) between January 1, 1974, and December 31, 2013; (2) have a histophatologically confirmed diagnosis of MB/CNS-PNET, (3) be diagnosed ≤ 20 years; and (4) have a minimum of 2 years observation time. In our sub-study, the survivors aged < 10 years at study start were not included due to challenges with test implementation [25], as were survivors unable to conduct tests due to severe cognitive and/or physical challenges after treatment.

Participants underwent validated taste and smell function tests (Burghart, Wendel, Germany) and a subjective evaluation of function. Information regarding each survivor’s diagnosis, treatment, and other relevant anamnestic information were gathered from the patient’s medical charts.

All tests were performed by the same dentist in an examination room at OUH.

### Subjective assessment of taste and smell

The survivors rated taste and smell function using a 0–10 numerical rating scale (NRS) when asked; “How well do you rate your taste/smell function?” Score 0 implied “no functional” sense, while score 10 implied “excellent function.” Participants were excluded if they were not able to rate their chemosensory function due to severe neurocognitive impairment.

### Test of taste function

Taste function was evaluated using taste strips (Burghart, Wedel, Germany), and the test took approximately 20 min for each participant. The test consists of filter-paper strips impregnated with four different concentrations of taste solutions of either sweet, salt, sour, or bitter. The concentrations for each of the tastes are as follows: sweet, 0.4, 0.2, 0.1, 0.05 g/ml sucrose; sour, 0.3, 0.165, 0.09, 0.05 g/ml citric acid; salty, 0.25, 0.1, 0.04, 0.016 g/ml sodium chloride; and bitter, 0.006, 0.0024, 0.0009, 0.0004 g/ml quinine hydrochloride [26, 27]. Strips with different taste qualities were randomly presented, one at a time from low to high concentration, to the anterior part of the tongue, and the participant were asked to identify the taste. Even if the participants did not sense a taste, they had to answer in a “forced-choice” procedure. The participant was asked to rinse their mouth with a sip of water between each strip. Total correct identification score was 16, with four correct answers for each taste quality.

### Normative values for taste

Evaluation of each participant’s taste-score was based on normative values [27] as instructed in the test protocol: normogeusia ≥ 9, hypogeusia < 9, and no sensation = complete ageusia (Burghart protocol) [27]. The taste function for each taste quality was assessed as normogeusia when ≥ 2 correct identifications of sweet, sour and salty, and ≥ 1 for bitter, while no taste sensation was regarded as ageusia of that specific taste quality. The survivors’ mean score of each taste quality were compared with normative values [12, 27].

### Test of smell function

Smell was assessed using the Sniffin’ Sticks test (Burghart, Wendel, Germany). The test consists of three subtests: threshold test (THR), discrimination test (DIS), and identification test (ID). Together THR, DIS, and ID yield a score, the “TDI-score,” which ranges between 1 and 48. Time spent administering all three tests was approximately 40 min. To minimize distractions, the investigator used odorless gloves and no perfumed body products.

### Threshold test (THR)

THR is performed in a “staircase procedure,” where the participant in each step is presented with three Sniffin’ pens (triplets). In each triplet, there are two pens without odor and one with odor, n-Butanol. The kit consists of 16 triplets, where triplet number 1 contains the pen with the highest concentration of the odor and triplet number 16 contain the pen with the lowest concentration. First, the participant is presented with the pen with the highest concentration, to be familiarized with the odor. The pen is held in front of the nostrils for a few seconds. Then the subject is exposed to the triplets from low to high concentration and asked to recognize the pen in each triplet with the odor. If the answer is correct, pens in the same triplet are shuffled and presented again. If the correct answer is given again, the examiner does a reversal of the “staircase procedure” until the subject is not able recognize the pen with the odor in a triplet. The test is over when the participant has been presented with seven staircase reversal steps and the final score is the mean value of the last four reversal steps.

### Discrimination test (DIS)

The aim of this test is to investigate if a subject can differentiate smells. The participant is presented with 16 different triplets of Sniffin’ pens. In each triplet, there are two pens with the same odor and one pen with a different odor. The task is to identify the pen with the different odor. The participant must provide an answer, “three-alternative forced choice.” Each pen is presented below the nostrils once for a few seconds with approximately 5 s between each pen in a triplet and approximately 30 s between each triplet. The score for DIS can range between 0 and 16.

### Identification test (ID)

ID consists of 16 pens with different odors. The aim of this test is to assess if the participant can identify everyday odors. Each pen contains a familiar odor and is held below the participant’s nose for a few seconds. The participant is asked to identify the odor by choosing one of the four alternatives for each pen, presented on a multiple-choice card. Even if the participant is not sure, a choice must be made. The interval between each pen is approximately 30 s. Maximum score of the ID is 16.

### Normative values for smell

Each participant’s TDI-score was classified based on normative data by Olesziewicz and coworkers (2019) [25], where a participant with a TDI-score of (1) ≤ 16.00 is regarded as having functional anosmia, (2) > 16.00 and < 30.75 is regarded as having hyposmia, (3) ≥ 30.75 and < 41.50 is regarded as having normosmia, and (4) ≥ 41.50 referred to as hyperosmia [25]. Additionally, the mean score of the three subtests and the mean TDI score of the 32 survivors were compared with normative data [25].

### Statistical analysis

Descriptive statistics were used for patient characteristics. Continuous variables were presented as mean with standard deviation (SD) and range in accordance with normative data [25, 27], and frequencies with proportion were presented for categorical variables. Analyses were performed using SPSS (IBM SPSS Statistics 27.0 for Windows, IBM Corp., Armonk, NY). Mean value of each taste quality and the mean scores of all three olfactory subtest and TDI score were compared with normative data [12, 25, 27] using MedCalc’s Comparison of means calculator: (https://www.medcalc.org/calc/comparison_of_means.php). A *p* value < 0.05 was considered statistically significant.

## Results

### Participants

**Fig. 1:**
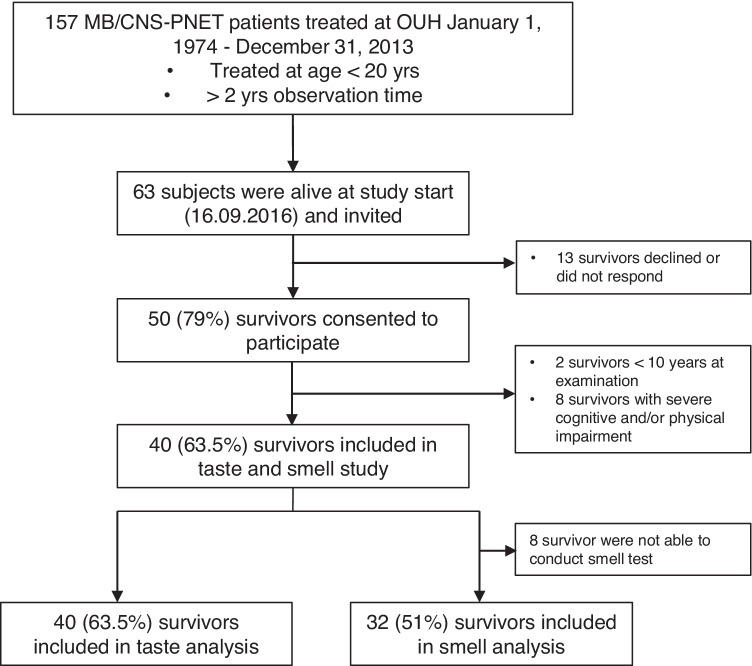
Flowchart of recruitment and inclusion of study population. MB, medulloblastoma; CNS-PNET, central nervous system supratentorial primitive neuroectodermal tumor; OUH, Oslo University Hospital

In total, 157 survivors treated for MB/CNS-PNET at OUH were identified during the selected study period. At study start, September 2016, 63 subjects were alive and invited. Figure 1 describes the recruitment and study inclusion of participants. Fifty (79%) of the survivors consented to participate in the multidisciplinary study. Two were excluded in this sub-study due to age < 10 years at the time of examination, and eight had severe cognitive and/or physical impairment. In total 40 (63.5%) survivors were included, and their characteristics are shown in Table 1. Eight out of 40 survivors were not able to conduct the smell test due to the complex olfactory test protocol. Hence, 32 (51%) survivors were included in the test of smell functions.

**Table 1.** Characteristics of long-term suvivors treated for MB/CNS-PNET at a young age *(n=40)*

Gender*, n (%)*	
Male	22 (55)
Age at treatment*, mean ± SD (yrs)*	8.4 ± 5.3 (range 0.2- 20)
Age at examination*, mean ± SD (yrs)*	28.9 ± 12.2 (range 10-52)
Time since treatment*, mean ± SD (yrs)*	20.5 ± 11.7 (range 3.5 - 40.4)
Tumor*, n (%)*	
MB	35 (87.5)
CNS-PNET	5 (12.5)
Treatment, n (%)	
Chemotherapy	32 (80)
Radiotherapy	35 (87.5)
Total taste strips score*, mean ± SD	10.1 ± 3.9 (range 2-16)
Normogeusia (>= 9)*, n (%)*	27 (67. 5)
Hypogeusia (< 9)*, n (%)*	13 (32.5)
Augesia of one or more taste quality*, n (%)	9 (22.5)
Sweet	1
Sour	5
Salt	4
Bitter	2
Taste function NRS score (0-10)*, mean ± SD*	8 ± 1.3 (range 5.5-10)
Total TDI score** in *n=32 , mean ± SD*	29.6 ± 3.2 (range 20-34.3)
Hyposmia (> 16-< 30.75)*, n (%)*	17 (53)
Normosmia (≥ 30.75-< 41.50)*, n (%)*	15 (47)
Smell function NRS score (0-10) in *n=32 , mean ± SD*	7.9 ± 1.5 (range 4.5-10)

*MB* medulloblastoma *CNS-PNET* central nervous system supratentorial primitive neuroectodermal tumor *NRS* numerical rating scale *TDI* sum score of treshold-, discrimination- , and identification test,*Scores based on Mueller et al. 2003 [27],**Scores based on Oleszkiewicz et al. 2019 [25]

### Taste function

The results of the 40 survivors who conducted the taste function test and were able to evaluate their own taste function, are listed in Table 1.

The mean value of total test score was 10.1 ± 3.9 (range 2–16). Thirteen (32.5%) participants scored < 9 and were diagnosed with hypogeusia. None of the subjects were diagnosed with complete ageusia, but 9 (22.5%) were ageusic for one or more taste qualities [27], with sour and salt as the most common ones (Table 1). The mean score of each taste quality is listed and compared with normative values [12, 27] in Table 2. MB/CNS-PNET survivors scored significantly lower on sweet, sour, and salt compared with normative data (Table 2). Based on NRS (0–10), the mean score of subjective evaluation of taste function was 8 ± 1.3 (range 5.5–10) (Table 1).

**Table 2.** Survivors *(n=40)* mean score of taste qualities compared with normative data [12, 27]

	Normative data*,* *mean* *(SD)*	MB/CNS-PNET survivors*,* *mean (SD)*	*p* value
Sweet	3.3 (0.8)	2.9 (1.2)	0.035
Sour	3.0 (0.8)	2.0 (1.1)	p < 0.001
Salty	3.1 (0.9)	2.4 (1.4)	0.003
Bitter	3.0 (1.1)	2.8 (1.2)	0.27

*MB* medulloblastoma *CNS-PNET* central nervous system supratentorial primitive neuroectodermal tumor

### Smell function

The results of objective olfactory tests and patients’ self-ratings are shown in Table 1. When TDI-scores of survivors were classified according to normative data [25], 17 (53%) survivors were diagnosed with hyposmia (Table 1). None of the subjects in our study were diagnosed with functional anosmia or hyperosmia. The mean scores of the three subtests and the mean TDI score in survivors compared with normative data [25] are presented in Table 3. We found the mean scores to be significantly lower (*p* < 0.0001) in survivors than in the normative data (Table 3).

**Table 3.** Survivors *(n=32)* mean score of smell tests compared with normative data [25]

	Normative data*, mean* *(SD)*	MB/CNS-PNET survivors*,* *mean* *(SD)*	*p* value
Treshold test (THR)	9.3 (3.0)	6.3 (1.8)	< 0.0001
Discrimination test (DIS)	13.0 (1.9)	11.2 (1.8)	< 0.0001
Identification test (ID)	13.6 (1.9)	12.1 (1.7)	< 0.0001
Total smell test score (TDI)	36.0 (4.2)	29.6 (3.2)	< 0.0001

*MB* medulloblastoma *CNS-PNET* central nervous system supratentorial primitive neuroectodermal tumor

## Discussion

This study is the first to evaluate both objective and subjective taste and smell function in long-term survivors after childhood MB/CNS-PNET. Hyposmia and hypogeusia were found in 53% of 32 survivors and 32.5% of 40 survivors, respectively. However, the patient reported rating of taste and smell function did not reflect the results of objective measurements.

Alterations of chemosensory function after treatment of brain tumors have been reported in only a few studies [20, 21]. Johannesen and coworkers (2002) found reduced taste function in three out of 33 long-term survivors of brain tumor treated ≥ 14 years (median time since treatment was 13.1 years), using qualitative examination of taste by identification of the four basic taste qualities [20]. However, comparison with our results is difficult since they did not describe the test protocol and how they evaluated the results [20]. Layrer and coworkers (2014) reported a relatively high degree of taste and smell disturbance 6 weeks after brain irradiation. They used a validated questionnaire, but no objective taste and smell measurement [21]. Since most brain tumor patients receive both RT and chemotherapy [2–4], it is hard to identify which of these treatment modalities may be of most significance when it comes to chemosensory disturbance [9, 10, 15, 23].

More than half (53%) of the participants in our study had a reduced smell function. In comparison, Cohen and coworkers (2014) reported only 3.9% subjects with smell dysfunction in a group of 51 survivors of different childhood cancers (including two MB survivors) with a mean time after treatment of 12.4 years [18]. IJpma and coworkers (2016) reported no difference in smell function in testicular cancer survivors compared to a control group [19]. The patient cooperation and attention needed throughout all three subtests of the Sniffin’ Sticks test [28] may be specifically challenging in brain cancer survivors. Even though survivors with severe cognitive and functional challenges were excluded in the present study, the relatively high prevalence of participants with reduced chemosensory function may be due to the vast variation in cognitive function after cancer treatment. Stadskleiv and coworkers (2020) have shown that cognitive function after treatment may vary considerable in MB/CNS-PNET survivors [24]. In another study, 60% of MB/CNS-PNET survivors had learning or memory problems compared to only 3% in a comparison group [1]. This is important since cognitive function may have a significant influence on olfactory testing, especially the identification and discrimination tests [29]. However, no such influence was observed on the olfactory threshold test [29], thereby emphasizing the importance of including a threshold test when assessing olfactory function in MB/CNS- PNET survivors. Additionally, there may be a cultural difference in odor detection, as showed in a Danish validation study of Sniffin’ Sticks [30]. They found that the original Sniffin’ Sticks (Burghart, Wendel, Germany) were not applicable in Denmark since several of the odors in the test were unfamiliar to the population [30].

Self-rating of olfactory function has been shown to have low reliability even in healthy subjects [31]; thus a validated objective measurement is recommended when assessing smell function [31, 32]. The participants in our study recorded a mean score of 7.9 ± 1.5 in self-evaluation of smell function, which did not reflect the results of the objective measurements. A similar discrepancy was found by Gurushekar and coworkers (2020) on HNC patients, prior to RT and up to 3 months after RT, using objective measurements and a questionnaire. The patients themselves did not notice smell dysfunction even though there was a significant reduction in olfactory function during RT [33]. The use of a validated patient-reported questionnaire [21, 33] would have gathered more profound information on the survivors’ subjective evaluation of chemosensory functions in our study.

In the present study, 32.5% of survivors were diagnosed as hypogeusic. This is in line with the results reported in the study on survivors of different childhood cancers by Cohen and coworkers (2014), where they found 27.5% with taste dysfunction using a 25 sample sipping test [18]. In the study by IJpma and coworkers (2016), impaired taste function was also found in testicular cancer survivors compared to a control group [19]. Cohen and coworkers (2014) and IJpma and coworkers (2016) both only reported reduced taste function with no reduction in smell function. This conflicts with other studies, in which solitary taste dysfunction is less frequent than smell impairment [10, 34–36]. Most often patients complaining of taste impairment have an olfactory deficit [10, 34–36].

None of the participants in our study was found to be complete ageusic. This is in accordance with results from other studies showing that complete ageusia is a rare condition [34, 37]. To differentiate “objectively” between hypogeusic and ageusic is difficult as revealed by Falk and coworkers (2013). Thus, the use of taste strips may be limited to differentiate between “healthy” and “non-healthy” subjects [37]. It should be mentioned that clinical assessment of taste function needs a multifactorial approach including evaluation of the patient’s complaints and symptoms, local oral morphology (e.g. tongue papillae), infections, saliva function, dental status, and use of any medication [10, 14]. 

Compared with normative data [27], the MB/CNS-PNET survivors in our study showed a significant lower value for the taste qualities sweet, sour, and salt. When the taste function results in a study on breast cancer patients were compared with normative data, only a significant lower value of sour quality on the left side of the tongue was found [12]. In our study, 22.5% of the survivors where ageusic to one or more taste qualities, with sour and salt being the most frequent quality lost. In HNC survivors, salt was also found to be one of the most impaired taste qualities, in addition to bitter [38]. Additionally, Barbosa da Silva and coworkers (2019) found that RT affected sweet, bitter, and sour sensitivities in HNC patients [15]. Impaired taste qualities may affect diet and nutritional status. A reduced intensity of different taste qualities, for instance, salt, may influence on nutritional behavior and may be associated with increased body mass index [39]. There may be genetic variations in taste receptors that may influence diet and nutritional behavior and risk of different diseases [40, 41]. In a Caucasian population, 25% was found to be non-tasters of compounds containing the thiocyanate group responsible for bitter taste [40, 42]. There is also a risk for misidentifying a taste quality, referred to as “taste confusion” [43]. In a study on 1000 participants with different health status sour-bitter confusion was reported to be the most common, while confusion involving the sweet quality was rare [43].

A strength of the present study was the use of objective validated tests for both taste and smell function and the inclusion of all three subtests for the evaluation of smell function. Additionally, the study population was homogeneous and relatively large compared to other studies in the literature, and the study had an exceptionally long mean time since treatment of over 20 years. An important limitation is the lack of a matched control group. Due to the wide spread in participants’ age at study start, the participants were not divided into age and gender groups when the results were compared with normative data. There is a significant correlation between taste function and age and gender, showing decreased taste with age and women exhibiting higher taste score than men regardless of age [26]. Furthermore, reduced olfactory function may be due to aging [14]. However, the main drop in olfactory identification ability occurs in the sixth and seventh decades of life [14], and none of our participants was in that age group. Our cross-sectional study provides information regarding prevalence of taste and smell function; however it does not assess how chemosensory function may change over time in relation to treatment. Unfortunately, our study population had no baseline test of taste and smell function. There may be a bias to our study that taste and smell function was reduced even before treatment started and a baseline test of chemosensory function is recommended in future studies.

In conclusion, a high prevalence of taste and smell impairment was found in survivors of childhood MB/CNS-PNET many years after treatment. Interestingly, most survivors did not report impaired function themselves. Nonetheless, reduced taste and smell function may still have severe impact on everyday life including diet, health, and risk of nutrition-related diseases. The medical team treating these patients should have knowledge and be aware of these possible long-term effects.

## Data and material availability

Can be available from corresponding author if requested.

## Supplementary information

Below is the link to the electronic supplementary material.

Supplementary file1 (DOCX 12 KB)

## Data Availability

Not applicable.
