# Supplementary material for: Taste and smell function in long-term survivors after childhood medulloblastoma/CNS-PNET
Source: Support Care Cancer. 2022 Apr 15;30(7):6155–62. doi: 10.1007/s00520-022-07048-9 (PMC9135811; doi:10.1007/s00520-022-07048-9)
Supplement: Supplementary file 1 — Supplementary file1 (DOCX 12 KB) [file 520_2022_7048_MOESM1_ESM.docx]

**Fig. 1** Flowchart of recruitment and inclusion of study population

Abbreviations: *MB* medulloblastoma, *CNS-PNET* central nervous system supratentorial primitive neuroectodermal tumor, *OUH* Oslo University Hospital
